# Mutant Huntingtin Derails Cysteine Metabolism in Huntington’s Disease at Both Transcriptional and Post-Translational Levels

**DOI:** 10.3390/antiox11081470

**Published:** 2022-07-27

**Authors:** Bindu D. Paul, Juan I. Sbodio, Solomon H. Snyder

**Affiliations:** 1 Department of Pharmacology and Molecular Sciences, Johns Hopkins University School of Medicine, Baltimore, MD 21205, USA; ssnyder@jhmi.edu; 2 Department of Psychiatry and Behavioral Sciences, Johns Hopkins University School of Medicine, Baltimore, MD 21205, USA; 3 The Solomon H. Snyder Department of Neuroscience, Johns Hopkins University School of Medicine, Baltimore, MD 21205, USA; jsbodio@yahoo.com; 4 Lieber Institute for Brain Development, Baltimore, MD 21205, USA

**Keywords:** Huntington’s disease, cysteine, cystathionine γ-lyase, transsulfuration

## Abstract

Cysteine is a semi-essential amino acid that not only plays an essential role as a component of protein synthesis, but also in the generation of numerous sulfur-containing molecules such as the antioxidant glutathione and coenzyme A. We previously showed that the metabolism of cysteine is dysregulated in Huntington’s disease (HD), a neurodegenerative disorder triggered by the expansion of polyglutamine repeats in the protein huntingtin. In this study, we showed that cysteine metabolism is compromised at multiple levels in HD, both transcriptional and post-translational. Accordingly, restoring cysteine homeostasis may be beneficial in HD.

## 1. Introduction

Huntington’s disease (HD) is a neurodegenerative disease that is caused by the expansion of CAG repeats, encoding polyglutamines, in the gene *huntingtin* [1]. Symptoms of HD include choreiform, involuntary movements, muscle wastage, impaired motor and executive functions, as well as psychiatric disturbances [2,3,4]. Mutant huntingtin (mHtt) misfolds and aggregates, forming oligomers and multimers that disrupt almost all cellular processes including transcription, translation, DNA replication, repair, nucleocytoplasmic transport, amino acid metabolism, and stress responses [5,6,7,8,9,10,11,12]. Presently, there is no cure for HD and the existing treatments only target the disease’s symptoms. Thus, there is an urgent need to elucidate the molecular mechanisms of pathogenesis, which can prove challenging, as mHtt affects a plethora of physiological processes.

Some of the molecular hallmarks of HD are elevated oxidative stress, mitochondrial dysfunction, and redox imbalance, which are intimately linked to each other. We and others have previously shown that oxidative stress in HD is linked to aberrant cysteine metabolism [13,14,15]. Apart from its indispensable role in protein synthesis, cysteine is the precursor of the antioxidant glutathione (GSH) and several sulfur-containing molecules such as cysteamine, taurine, coenzyme A, iron-sulfur cluster proteins, and lanthionine [16]. Additionally, cysteine serves as the substrate for production of the gaseous signaling molecule, hydrogen sulfide (H_2_S), which participates in several cellular processes by eliciting a post-translational modification termed persulfidation/sulfhydration [17,18,19].

**Figure 1:**
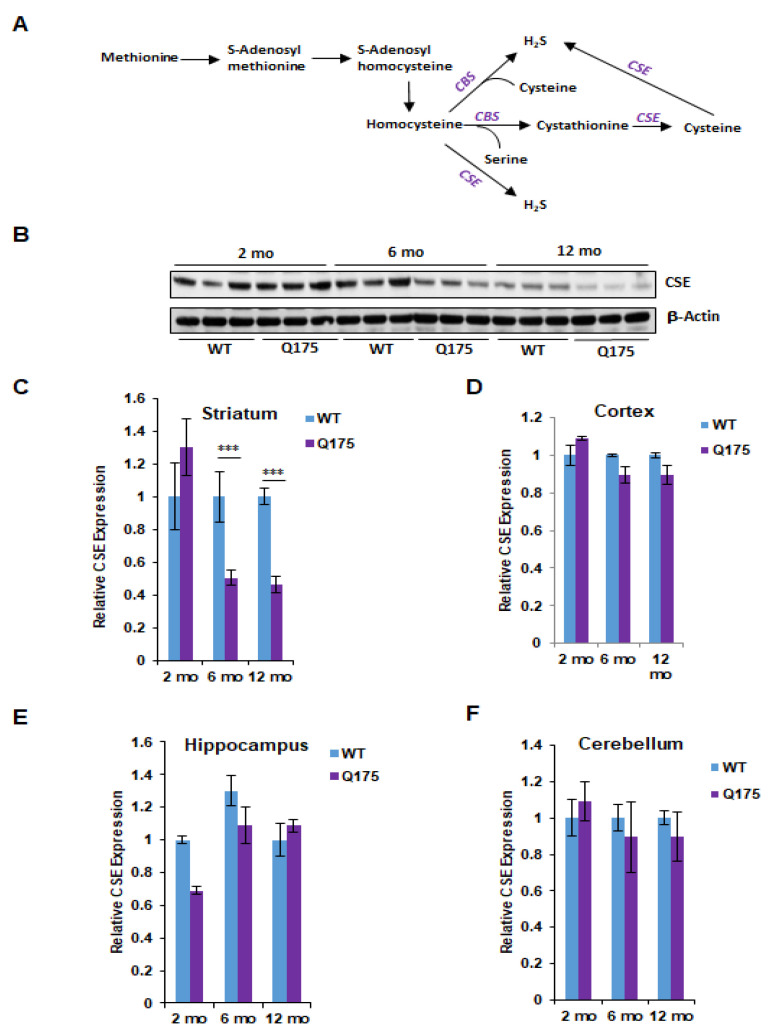
Expression of cystathionine γ-lyase (CSE) in the Q175 model of Huntington’s disease. (**A**) The transsulfuration pathway via which cysteine and hydrogen sulfide (H_2_S) is generated. Transsulfuration refers to the transfer of sulfur from homocysteine (which is derived from dietary methionine) to cysteine. Cystathionine β-synthase (CBS) condenses serine with homocysteine to generate cystathionine, which is acted on by CSE to produce cysteine. Cysteine and homocysteine serve as substrates of CSE or CBS to generate H_2_S. (**B**) Expression of CSE at 2, 6, and 12 months in the striatum, the region most profoundly affected in HD, in Q175 mice and their wild-type counterparts. (**C**) Quantification of B. (**D**–**F**) Expression of CSE at 2, 6, and 12 months in the cortex, hippocampus and cerebellum. *n* = 3, SEM. *** p < 0.001.

Cysteine is generated via the reverse transsulfuration pathway by cystathionine γ-lyase (CSE), which is the major enzyme that generates cysteine in mammals (Figure 1A). Although cysteine is a semi-essential amino acid, for mice lacking CSE, it is an essential amino acid. Thus, when *Cth*^−/−^ mice are placed on a cysteine-free diet, they rapidly lose weight and die within two weeks, unless external cysteine is provided [9,20,21]. We and others have previously shown that dysregulation of cysteine and H_2_S metabolism occurs in several neurodegenerative diseases such as Parkinson’s disease (PD), Huntington’s disease (HD), Alzheimer’s disease (AD), amyotrophic lateral sclerosis (ALS), and spinocerebellar ataxia (SCA) [9,11,13,22,23,24,25], and in neurodevelopmental disorders such as Down syndrome [26,27]. In HD, the disruption of cysteine balance occurs due to the inhibitory effects of mHtt on CSE expression by its transcription factors, SP1 and ATF4. The dysregulation of the transsulfuration pathway was also postulated in an earlier study, which reported the interaction of CBS with mHtt [28]. More recently, we showed that the dysregulation of transsulfuration occurs during aging, which is a risk factor for neurodegeneration [29].

In this study, we found that in addition to regulation at the transcriptional level, the activity of CSE is also regulated at the post-translational level in HD.

## 2. Materials and Methods

### 2.1. Cell Culture

HEK-293 cells were grown in a humid atmosphere of 5% CO_2_ at 37 °C in DMEM supplemented with 10% (*v*/*v*) FBS, l-glutamine (2 mm), penicillin (100 units/mL), and streptomycin (100 µg/mL).

### 2.2. Immunoprecipitations

The cells were lysed on 1.5 mL tubes and placed on ice in a buffer containing 50 mm Tris·HCl, pH 7.4, 150 mm NaCl, 1% (*v*/*v*) Triton X-100, 1 mm EDTA, 1 mm PMSF, 10% (*v*/*v*) glycerol, and protease inhibitor tablet (Millipore, Burlington, MA, USA), and centrifuged at 16,000× *g* for 15 min and supernatant collected. In total, 500 μg of the extract in a total volume of 1 mL was incubated with anti-CSE antibodies [30] overnight at 4 °C on a rotator. Next, 25 μL of ProteinA/G agarose was added and the samples incubated overnight on a rotary shaker (Calbiochem, San Diego, CA, USA). The lysates were spun down on the next day, washed three times with the IP buffer, eluted in 1x LDS buffer, boiled at 95 °C for 5 min, centrifuged for 3 min at 16,000× *g,* and supernatant was loaded. About 5% of the original lysate was used as the input.

### 2.3. Preparation of Tissue Homogenates

For the activity assays of CSE, the tissue was homogenized in a buffer containing 50 mM phosphate buffer, pH 7.4 containing 0.5% Triton X 100 and protease inhibitors on ice for 15 min, followed by centrifugation at 16,000× *g* for 15 min and recovery of the supernatant. For assaying CBS activity, the samples were processed as described earlier [31]. The homogenate anti-CSE antibodies were previously described [30].

### 2.4. Western Blotting

For Western blots, lysates were normalized for total protein content using Protein Assay Dye Reagent (Bio-Rad). The samples were prepared by adding 1× of the final concentration of NuPAGE LDS Sample Buffer (Invitrogen), followed by incubation at 95 °C for 5 min. Subsequently, the protein samples were resolved on a mini NuPAGE 4–12% Bis-Tris gel (Thermo Fisher, Scientific, Waltham, MA, USA) in the presence of a 1× NuPAGE MES SDS running buffer (Thermo Fisher, Scientific, Waltham, MA, USA). The proteins were then transferred to Immobilon-FL (Millipore). The membranes were blocked for 1 h at room temperature with 5% BSA in TBS, followed by incubation with the indicated primary antibodies. Horse peroxidase-conjugated secondary antibodies were used for detection with SuperSignal West Pico chemiluminescence reagent (Thermo Fisher, Scientific, Waltham, MA, USA). The detection of CBS was performed using commercial antibodies from Santa Cruz.

### 2.5. H_2_S production Assays

H_2_S production was measured according to previously published protocols [30]. For assays involving CSE, 10 mM L-cysteine was used as the substrate. CBS does not utilize cysteine alone for H_2_S production, hence for the assays of CBS, 10 mM L-cysteine and 15 mM L-homocysteine (the preferred substrates) were utilized.

### 2.6. Cysteine Production Assay

The cysteine formed by CSE was quantified using the ninhydrin assay. The ninhydrin reagent was prepared by dissolving 250 mg of ninhydrin in 6 mL of glacial acetic acid and 4 mL of 12 M HCl. The samples (200 µL) were mixed with 60 µL of 6% perchloric acid, and the precipitated protein was removed by centrifugation. Next, 200 µL of the supernatant was mixed with 200 µL of glacial acetic acid and ninhydrin, then boiled for 10 min, cooled on ice, and mixed with 95% ethanol to a final volume of 1 mL. The absorbance was measured at 560 nm.

### 2.7. Statistical Analysis

Statistical analyses were performed using Microsoft Excel. A value of *p* < 0.05 was considered statistically significant. Statistical analyses were conducted as appropriate using an unpaired Student’s *t*-test. Each bar represents the mean ± standard error of the mean (SEM).

## 3. Results

### 3.1. Expression of Cystathionine γ-Lyase and Cystathionine β-Synthase in HD

We examined the expressions of CSE and CBS in several regions of the brains of Q175 mice at the RNA and protein levels at different stages of disease progression. To evaluate early vs. medium-term vs. advanced disease, we, respectively, employed 2, 6, and 12 months as the age groups for analysis. In terms of the brain regions, the striatum is the site of the greatest degeneration in human HD. In mouse models, other parts of the brain may be affected to a similar extent, though the distribution of damage varies with the specific HD model. We certainly aimed to focus upon the striatum. The cerebellum was of interest too because, like the striatum, the cerebellum regulates motor activity. The hippocampus and cerebral cortex are of importance because of their roles in cognition, learning, and memory, which are substantially impaired in HD. For these reasons, we analyzed these four regions of the brain.

We first focused on the protein levels for CSE and CBS in the brain regions at the three ages. The most striking alterations were evident in CSE levels of protein in Q175 striatum at 6 and 12 months when the levels were reduced by about 50%, while at 2 months, there was no change (Figure 1B,C). In the cortex, hippocampus and cerebellum, CSE protein levels did not significantly differ between wild-type and Q175 mice at any of the three ages (Figure 1D–F).

**Figure 2:**
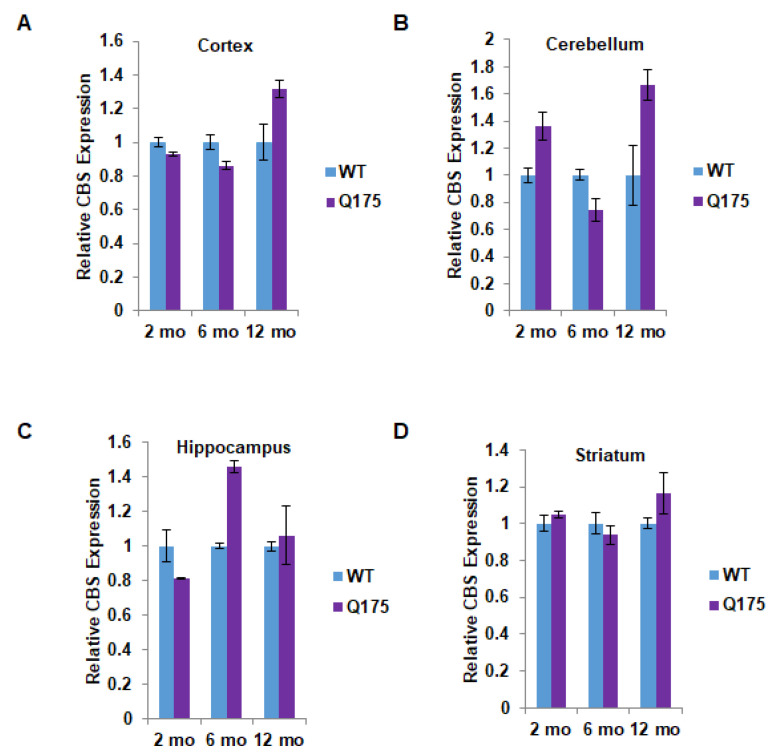
Quantitation of cystathionine β-synthase (CBS) expression in the Q175 model of Huntington’s disease. (**A**) Expression of CBS at 2, 6, and 12 months in the cortex in Q175 mice and their wild-type counterparts. (**B**–**D**) Expression of CBS at 2, 6, and 12 months in the cerebellum, hippocampus, and striatum in Q175 mice. *n* = 3, SEM.

The protein disposition of CBS markedly differed from that of CSE. Thus, at no point in time, nor in any brain region, was the CBS protein level significantly decreased in Q175 mice (Figure 2A–D). In most instances, there was no difference between wild-type and Q175 mice in terms of CBS protein levels. Surprisingly, there appeared to be a significant increase in CBS protein levels in the cerebellum and hippocampus of Q175 mice. In the cerebellum, the increase of about 60% was evident only at 12 months (Figure 2B), while in the hippocampus, a 40–50% increase was apparent at 6 but not at 2 or 12 months (Figure 2C).

CBS does not occur in neurons in the brain [31,32]. It is largely concentrated in glia, especially astrocytes. With neural degeneration, there is often an associated reactive proliferation of astrocytes and other glia, especially microglia. Perhaps the elevations that we observed relate to such proliferation. By contrast, our unpublished studies of CSE localization in normal mouse brains indicate the existence a prominent neuronal disposition. The previous studies from our laboratory revealed that CSE depletion in HD may reflect mutant huntingtin binding to and inhibiting the transcription factor specificity protein 1 (SP1), which is the transcription factor driving the basal expression of CSE. This mechanism may well-account for the loss of CSE protein in HD. The profound diminution of brain CSE levels in HD of the patient’s brain, R6/2 mice, and Q111 striatal cultures is maintained even when one corrects for the loss of neurons related to the overall cellular degeneration of the disease.

### 3.2. Decreased CSE and CBS Activities in Q175 Mice

**Figure 3:**
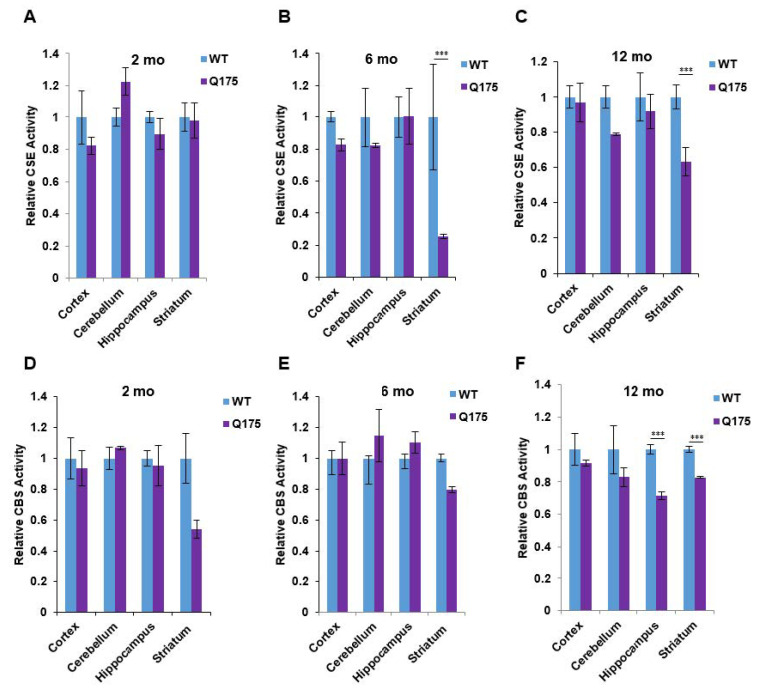
Enzymatic activities of cystathionine γ-lyase (CSE) and cystathionine β-synthase (CBS) in the Q175 model of Huntington’s disease. (**A**–**C**) Enzymatic activity of CSE at 2, 6, and 12 months in the cortex, cerebellum, hippocampus, and striatum in Q175 mice and their wild-type counterparts. H_2_S production from 10 mM L-cysteine in the presence of the cofactor, pyridoxal 5-phosphate (PLP at 250 mM), using the methylene blue assay (**D**–**F**). Enzymatic activity of CBS at 2, 6, and 12 months in the cortex, cerebellum, hippocampus, and striatum in Q175 mice and their wild-type counterparts. Activity was measured using the substrates for CBS (15 mM L-homocysteine and 10 mM L-cysteine) using the methylene blue assay. *n* = 3, SEM. *** p < 0.001.

We wondered whether the decreased protein level of CSE in the brain of Q175 mice may be associated with decreases in catalytic; and hence, we measured the protein levels of CSE and CBS in the forebrain regions at three ages (Figure 3). The most prominent alteration observed was a massive depletion of CSE activity in the Q175 striatum at 6 and 12 months, whereas the CSE activity in the cortex, cerebellum, and hippocampus in the Q175 mice was not altered (Figure 3B,C). By contrast, at 2 months, CSE activity in the Q175 mice was the same in all of the brain regions as in the wild-type mice (Figure 3A). In the case of CBS, decreased activity was observed in the striatum at all three ages (Figure 3D–F) along with a decrease in CBS activity in the hippocampus at 12 months (Figure 3F).

### 3.3. Huntingtin Interacts with CSE and Modulates Its Catalytic Activity

**Figure 4:**
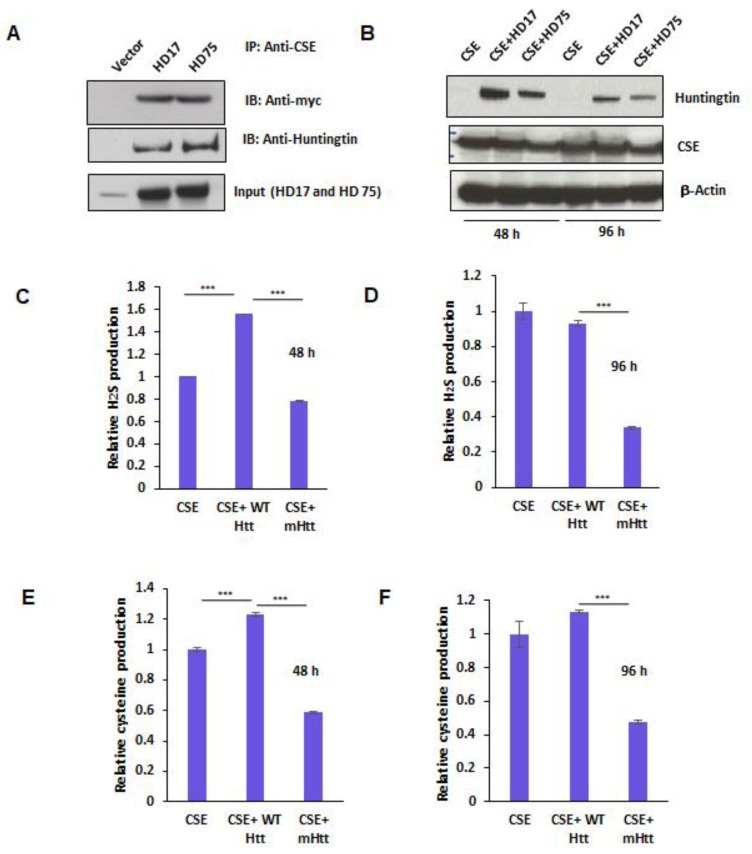
Mutant huntingtin (mHtt) binds cystathionine γ-lyase (CSE) and inhibits its catalytic activity. (**A**) Interaction of CSE with huntingtin. HEK293 cells were transfected with constructs encoding myc-tagged CSE and either HD17 or HD75 for 24 h and immunoprecipitation conducted using anti-myc antibody. CSE bound both wild-type and mutant Htt. (**B**) Expressions of CSE, WTHtt, and mHtt at 48 and 96 h post-transfection as determined by Western blotting. (**C**,**D**) Activity of CSE at 48 and 96 h, respectively, as measured by assaying H_2_S production from 10 mM L-cysteine in the presence of the cofactor, pyridoxal 5-phosphate (PLP at 250 mM), using the methylene blue assay. (**E**,**F**) Enzymatic activity of CSE at 48 and 96 h respectively, as measured by assaying cysteine production from cystathionine in the presence of the cofactor, pyridoxal 5-phosphate (PLP at 250 mM), using the ninhydrin assay. *n* = 3, SEM, *** p < 0.001.

In order to further characterize the influence of mHtt on CSE, we tested whether mHtt binds to CSE. We transfected plasmids encoding myc-tagged CSE with either full-length wild-type Htt, wtHtt (HD17), or mHtt (HD75) into HEK293 cells for 24 h and conducted immunoprecipitation assays. Both wtHtt and mtHtt bound to CSE, with mtHtt showing stronger binding (Figure 4A). Next, we assayed the effect of this interaction on the catalytic activity of CSE. Accordingly, we transfected CSE alone or in combination with either HD17 or HD75 for 48 and 96 h to allow for mHtt aggregation. The lysates from the transfected cells were prepared, and the activity of CSE was determined by measuring H_2_S and cysteine production. In parallel, the expressions of CSE and huntingtin were monitored (Figure 4B). The production of cysteine was measured using a ninhydrin assay, and H_2_S production using the substrate, cysteine and pyridoxal 5-phosphate (PLP), as described earlier [13,33]. Interestingly, we observed that at 48 h, wtHtt stimulated the activity of CSE, both in terms of H_2_S as well as cysteine production, while mHtt inhibited it (Figure 4C,E). Additionally, at 96 h, the inhibitory effect of mHtt on both cysteine and H_2_S production was more pronounced than at 48 h (Figure 4D,F). Thus, mHtt acts to derail cysteine metabolism by inhibiting the catalytic activity of CSE.

## 4. Discussion

The principal finding of this study is that the transsulfuration pathway, which plays central roles in neuroprotective processes, is disrupted at multiple levels in HD. Our prior studies have identified a major decrease in CSE levels in human HD brains, in the brains of R6/2 HD mice, and in Q111 striatal cell lines, which are associated with impaired stress responses [9,11,13]. Additionally, other groups have reported a suboptimal expression and activity of the transporters for cystine and cysteine, respectively [15,34]. We have also shown that cysteine homeostasis is linked to stress responses such as the oxidative stress response, organellar stress responses such as the ER stress response, and to the Golgi stress response [9,11,16,35,36]. CSE is an inducible protein and is highly sensitive to stress stimuli, with its response being compromised in HD. Because cysteine produced by CSE is a building block of the cellular antioxidant glutathione, and cysteine is the precursor of several sulfur containing neuroprotective molecules, any disruption of its metabolism can have deleterious effects. The clinical relevance of these findings is evident from the ability of cysteine repletion utilizing *N*-acetylcysteine (NAC) to reverse the behavioral abnormalities of R6/2 mice [13]. Independent studies also validated the neuroprotective effects of NAC in HD using other mouse models such as R6/1, where mitochondrial dysfunction and behavioral abnormalities were ameliorated by NAC treatment [37].

**Figure 5:**
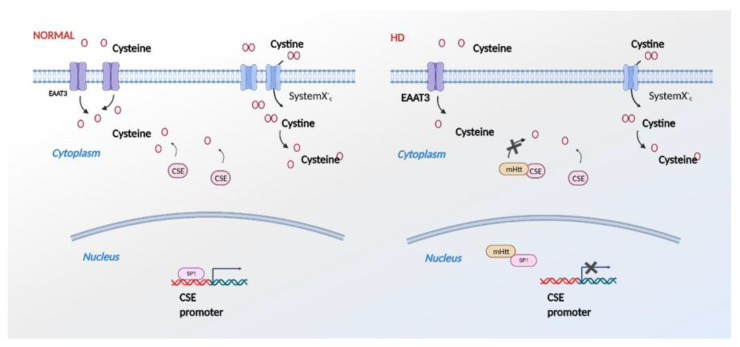
Effects of mutant huntingtin (mHtt) on cysteine metabolism. In HD (right panel), mHtt binds specificity protein 1 (SP1) and inhibits transcription of CSE. mHtt also impairs import of cystine and cysteine by the system Xc^-^ and excitatory amino acid transporter 3 (EAAC1/EAAT3), creating an overall cysteine deficit. In addition to these effects, mHtt binds directly to CSE and inhibits its catalytic activity.

In HD, cysteine metabolism is attenuated not only at the level of its biosynthesis at the transcriptional level by the influence of mHtt on SP1 and ATF4, but also by the impaired import of both cysteine and cystine. In addition to these modes of action, mHtt binds CSE and inhibits its catalytic activity (Figure 5). Thus, mHtt acts at multiple levels, consistent with reports in the literature. An interesting observation in this study was that wtHtt stimulated activity in the CSE, which is an area worth investigating further. The functions of normal, wild-type huntingtin are still being elucidated and, thus, a deeper insight into the interaction and novel activities of huntingtin would improve our understanding of the in vivo actions of this multi-faceted protein. This study revealed the interaction between huntingtin and cysteine homeostasis. Another point to be noted is that the length of the polyglutamine tract is high, around 175 in the mouse model and 75 in the construct, with these expansions being longer than the typical lengths found in human HD. However, in our previous study, we demonstrated that in human HD too, expression of CSE is decreased, and the magnitude of the depletion correlates with the severity of the disease [13]. A systematic study analyzing the effects of the length of the polyglutamine tract would be an area for future investigation. The new finding in this study was that mHtt interacts with CSE and decreases its catalytic activity, adding another layer of complexity to the regulation of the transsulfuration pathway in HD.

The dysregulation of cysteine homeostasis has been observed in aging, and cysteine deficits have been reported to cause structural and behavioral deficits in mice lacking EAAC1 [29,38]. Aging is a major risk factor for neurodegeneration and, as cysteine metabolism is suboptimal in a wide variety of these conditions, upregulating the transsulfuration pathway as a whole may be beneficial. This may involve the modulation of the expression of CSE and CBS, or stimulation of their catalytic activities by small molecules. Accordingly, screening for regulators of the transsulfuration pathway, which is fine-tuned at multiple levels, may be beneficial in not only HD, but also in diseases involving a cysteine deficit.

## Data Availability

Data is contained within the article.
